# Comparison of Defined Course Doses (DCD_vet_) for Blanket and Selective Antimicrobial Dry Cow Therapy on Conventional and Organic Farms

**DOI:** 10.3390/ani9100707

**Published:** 2019-09-20

**Authors:** Clair L. Firth, Annemarie Käsbohrer, Christa Egger-Danner, Klemens Fuchs, Beate Pinior, Franz-Ferdinand Roch, Walter Obritzhauser

**Affiliations:** 1 Unit of Veterinary Public Health & Epidemiology, Institute of Food Safety, Food Technology and Veterinary Public Health, University of Veterinary Medicine, 1210 Vienna, Austria; annemarie.kaesbohrer@vetmeduni.ac.at (A.K.); beate.pinior@vetmeduni.ac.at (B.P.); Franz-Ferdinand.Roch@vetmeduni.ac.at (F.-F.R.); w.obritzhauser@dairyvet.at (W.O.); 2 ZuchtData EDV-Dienstleistungen GmbH, 1200 Vienna, Austria; egger-danner@zuchtdata.at; 3 Data, Statistics and Risk Assessment, Austrian Agency for Health and Food Safety (AGES), 8010 Graz, Austria; klemens.fuchs@ages.at

**Keywords:** antibiotics, dry cow therapy, cattle, prudent use, dairy, veterinary

## Abstract

**Simple Summary:**

The general public is increasingly concerned about using antibiotics to treat animals in food production systems. It is vital that sick animals should be treated and that veterinarians should have treatment options available to prevent unnecessary suffering. Nevertheless, farmers and veterinarians are working together to reduce antibiotic use. In Austria, antibiotics can only be obtained from veterinarians and any antimicrobials dispensed must be reported to the authorities. This study aimed to compare antibiotic use on dairy farms, using a standardised unit, the Defined Course Dose. The most common bacterial infection in dairy cows is mastitis (udder infection). For decades, the use of antibiotic treatments to prevent or cure mastitis during the dry period (approximately 4–8 weeks when cows are not milked) has been routine. However, the need to reduce antibiotic use has seen farmers and veterinarians consider a more selective use of these drugs, only using them in cows proven to have bacterial infections, rather than treating all cows in a “blanket” approach. This study determined that farmers choosing a selective approach used fewer antibiotics overall than those using blanket treatment. No difference was found between conventional and organic dairy farms with respect to antibiotic use at drying off.

**Abstract:**

Antimicrobial use in livestock production is a controversial subject. While antimicrobials should be used as little as possible, it is still necessary, from both an animal health and welfare point of view, to treat infected animals. The study presented here aimed to analyse antimicrobial use on Austrian dairy farms by calculating the number of Defined Course Doses (DCD_vet_) administered per cow and year for dry cow therapy. Antimicrobial use was analysed by production system and whether farmers stated that they used blanket dry cow therapy (i.e., all cows in the herd were treated) or selective dry cow therapy (i.e., only cows with a positive bacteriological culture or current/recent history of udder disease were treated). A statistically significant difference (*p* < 0.001) was determined between antimicrobial use for blanket (median DCD_vet_/cow/year: 0.88) and selective dry cow therapy (median DCD_vet_/cow/year: 0.41). The difference between antimicrobial use on conventional and organic farms for dry cow therapy as a whole, however, was not statistically significant (*p* = 0.22) (median DCD_vet_/cow/year: 0.68 for conventional; 0.53 for organic farms). This analysis demonstrates that selective dry cow therapy leads to a lower overall use of antimicrobials and can assist in a more prudent use of antimicrobials on dairy farms.

## 1. Introduction

The general public increasingly considers the use of antimicrobials in livestock production excessive and a risk to human health [1,2,3]. Although dairy production uses a relatively low amount of antimicrobial substances compared to other livestock industries such as pig production [4,5], it has long been known that udder diseases make up the largest proportion of antimicrobial use in this sector [6,7,8]. Since the introduction of the five-point plan for mastitis prevention in 1966 [9], the use of antimicrobial dry cow therapy at drying off in all cows has been a routine herd health procedure worldwide. Indeed, treating cows with persistent high somatic cell counts (SCC) over an extended period with antimicrobials is often the best way to ensure bacterial cure [10,11,12,13,14]. Dairy cows are often already infected with mastitis pathogens at the time of drying off, as shown by, for example, Kiesner and colleagues in a German study where 38.4% of 250 cows from five different farms had intramammary infections (excluding minor pathogens) at this time [15]. This study also demonstrated that cows receiving antimicrobial dry cow therapy (aDCT) had a significantly higher cure rate (86.6%) than cows that did not receive antimicrobial treatment (59.2%), confirming the importance of treating udder disease at this time [15]. Similarly, an Estonian study reported extremely high elimination rates of mastitis pathogens during the dry period when cows were treated with cloxacillin-containing dry cow intramammary injectors, namely 100% for *Streptococcus uberis* and 93.6% for *Staphylococcus aureus* [12]. While the antimicrobial treatment of infected cows is therefore essential, a number of countries have attempted to implement a more prudent use of antimicrobials in the dairy sector and some countries such as Finland, the Netherlands and Denmark have effectively banned the prophylactic use of antimicrobials at drying off for all cows (blanket dry cow therapy) if their infection status is not known [16,17,18].

Unlike their counterparts in North America, organic farmers in the European Union are permitted to use antimicrobials to treat infected animals, however, there are a number of restrictions on their application [19,20]. In particular, the preventive use of medication in healthy animals on organic farms is prohibited and organically produced cattle are not permitted to be treated with “chemically synthesised allopathic” medicines more than three times a year [19]. As such, organic dairy farmers in Europe may only use antimicrobial dry cow therapy if they can prove that the cow’s udder is infected and needs such treatment; this is usually done by means of bacteriological culture of milk samples prior to drying off or based on health records for each cow’s lactation. These restrictions on antimicrobial use in the organic sector have led to the assumption that the overall use of antimicrobial agents is lower on these types of farms than on those managed according to conventional practices [21].

Regardless of production system, the use of antimicrobial substances in food-producing animals is strictly controlled in Austria. Veterinary antimicrobials can only be obtained from a veterinarian and are not available for sale over the counter. Farmers and veterinarians are required to keep treatment records for all food-producing animals in accordance with local and EU laws. Furthermore, antimicrobials may only be dispensed to farmers who are members of the Austrian Animal Health Service and who have completed a training course in medicine administration. Since 2015, veterinarians have also been legally obliged to report all antimicrobials dispensed for use in food-producing animals to the relevant national authorities, however, they do not need to report antimicrobials administered themselves. The data presented here included all antimicrobial use on the study farms, as we collated antimicrobial use by the veterinarians themselves, as well as data on veterinary dispensing of antimicrobials to these farms. This process, therefore, provided a more complete picture of antimicrobial use than statutory reporting alone.

The aim of the current observational study was to analyse the differences in antimicrobial use, quantified by the Defined Course Dose (DCD_vet_) metric, between blanket and selective approaches to aDCT. To this end, we aimed to compare responses regarding blanket and selective dry cow therapy, as well as a variety of other farm management factors, given by both conventional and organic dairy farmers to an online questionnaire and to analyse actual antimicrobial use as recorded by herd veterinarians for dry cow therapy on these farms over a one-year period.

## 2. Materials and Methods

### 2.1. Participant Selection

The analysis presented here was part of a larger multidisciplinary study of antimicrobial use and farm management factors with respect to mastitis incidence on Austrian dairy farms. A total of 17 veterinary practices took part in this year-long observational study of veterinary treatment of udder disease as previously described [22,23]. Veterinarians were invited by the authors to participate and were then asked to nominate dairy farmers from their practice areas. No restrictions were made with respect to herd size. All dairy farms were required to be enrolled in the national milk recording service and the Austrian Animal Health Service. This analysis is, therefore, based on a convenience sample (i.e., a non–probability sampling method from a group of people that the researchers can easily contact) and is not necessarily representative of antimicrobial drying-off practices in the remainder of Austria. Furthermore, the authors did not know at the time of enrolment whether farms were run conventionally or organically, which is why the production groups included here are not split equally.

Both farmers and veterinarians completed informed consent forms and agreed to the use of their herd or treatment data being analysed within the realms of this study.

### 2.2. Ethics Committee Approval

Although this was an observational study of local veterinarians treating cattle at their own clinical discretion, the study protocol was discussed and approved by the institutional ethics and animal welfare committee of the University of Veterinary Medicine, Vienna (Ref. No. ETK-13/11/2015), in accordance with good scientific practice guidelines and national legislation.

### 2.3. Electronic Data Collection

As previously described with respect to the treatment of acute and chronic mastitis cases [22], antimicrobials used at drying off were collated from the practice software of participating veterinarians via an electronic interface with the online central cattle data network (run by the Austrian Federation of Cattle Breeders, ZAR, as described by Koblmüller and colleagues [24]). The data collection period ran from 1st October 2015 to 30th September 2016 (i.e., one milk performance reporting period).

In addition to statutory reports of all antimicrobials dispensed to farmers for use in food-producing animals, this observational study collected data on antimicrobials administered directly by veterinarians. Data were collected on all veterinary medicinal products, the identification number of the cattle treated and the disease indication recorded by the treating veterinarian. Herd veterinarians were solely responsible for choosing which antimicrobials to apply.

### 2.4. Calculation of Defined Course Doses per Cow and Year

Data were collated electronically from the practice software of 17 participating veterinary practices. Details on the data collected have been described elsewhere [22], and suffice to say, the form of medication (in this case, dry cow udder intramammary injectors), number of injectors, identification number of the cow, agricultural holding number and date of administration or dispensing were all recorded and converted into a Microsoft Excel spreadsheet for analysis.

According to the European Medicines Agency recommendations, four dry cow intramammary injectors were classed as one Defined Course Dose (DCD_vet_), regardless of antimicrobial active ingredient or concentration [25]. No adjustments were made for liveweight, as the DCD_vet_ with respect to dry cow therapy is calculated as antimicrobial treatment per udder (i.e., per cow) rather than per kilogram. The Austrian Federation of Cattle Breeders provided information on the number of cow production days within the one-year observational period (defined as the number of days an animal was in the herd), calving interval and replacement rates for each of the farms included in the present study. Using these data, the number of DCD_vet_/cow/year for each farm was corrected to allow for these factors (as shown below), in a similar manner to that used by other authors (e.g., More and colleagues in Ireland [26]). It was assumed that each cow that was dried off with antimicrobials received four dry cow intramammary injectors (as cows with blind quarters were not reported).

The number of Defined Course Doses (nDCD_vet_) administered on each farm was calculated using the DCD_vet_ values assigned to dry cow intramammary injectors by the European Medicines Agency [25] as follows:

(1) $$nDCDvet=\frac{\Sigma \;\left(total\;number\;of\;dry\;cow\;injectors\;used\;on\;this\;farm\right)}{4}$$

To calculate the nDCD_vet_/cow/year for each farm, the following formula was used:

(2) $$nDCDvet/cow/year=\frac{nDCDvet}{Production\;days\;on\;this\;farm}\times 365$$

The nDCD_vet_/cow/year for each farm was then multiplied using the following correction factor based on central cattle database figures for each study farm:

(3) $$Correction\;factor=\frac{Calving\;interval\;\left(d\right)}{365}\times \frac{100}{\left(100-replacement\;rate\;\left(\%\right)\right)}$$

### 2.5. Farmer Questionnaires

Farm management questionnaires were prepared and hosted on the online survey system, SoGoSurvey (SoGo Survey Inc, Herndon, VA, USA, www.sogosurvey.com). Questionnaires were sent via email (or post (A very small number of farmers did not provide their email address, and, for this reason, a total of 16 questionnaires were sent by post, with a pre-paid return envelope enclosed)) to 251 farmers, with reminders sent on a monthly basis to those farmers with email addresses. Extensive details on questionnaire design, question type and content have been published elsewhere [23]. Questions particularly relevant to the current study included the farmers’ self-declaration of their conventional or organic status, as well as the following question on DCT procedures (translated from the original German):

“Are your cows dried off with antimicrobials?” Possible answers: “Yes, all cows” (*included in this study as “blanket DCT”*); “Only symptomatic cows (altered milk, swollen udder, California mastitis test (CMT) positive, etc.)” (*included here as “selective DCT”*); “Only cows with a positive bacteriological culture” (*included here as “selective DCT”*) or “Antimicrobial DCT is not used”.

### 2.6. Statistical Analysis

Due to data distribution and group size, the non–parametric Wilcoxon rank sum test with continuity correction for unpaired samples was used to identify whether a significant difference existed between blanket and selective treatments overall, as well as between conventional and organic farms, with respect to dry cow therapy expressed in DCD_vet_/cow/year. Farmers who did not answer the questions relating to DCT or their production system or for whom antimicrobial use data were not available were excluded from the respective statistical analysis. A further five farms (from a total of 193) were in the process of converting from conventional to organic production and were also excluded from the analysis of production system as the number of cases reported was too small to allow for a meaningful analysis. These farms were included in the overall analysis of blanket versus selective DCT.

To compare all four possible treatment groups (i.e., conventional and organic farms with respect to blanket or selective DCT), a non–parametric Kruskal–Wallis rank sum test was applied, followed by a pairwise test for multiple comparison of mean rank sum (Dunn’s test), alpha-adjusted with Bonferroni (B) and Benjamini–Hochberg (BH) [27]. To better assess the limited validity of the comparison between small and large sample sizes, both alpha adjustments were done (i.e., the more conservative B correction and less conservative approach with BH). Data were considered significant at *p* ≤ 0.05. Statistical analyses were carried out using the R software package for statistical computing (www.r-project.org).

## 3. Results

### 3.1. Questionnaire Respondents

**Figure 1:**
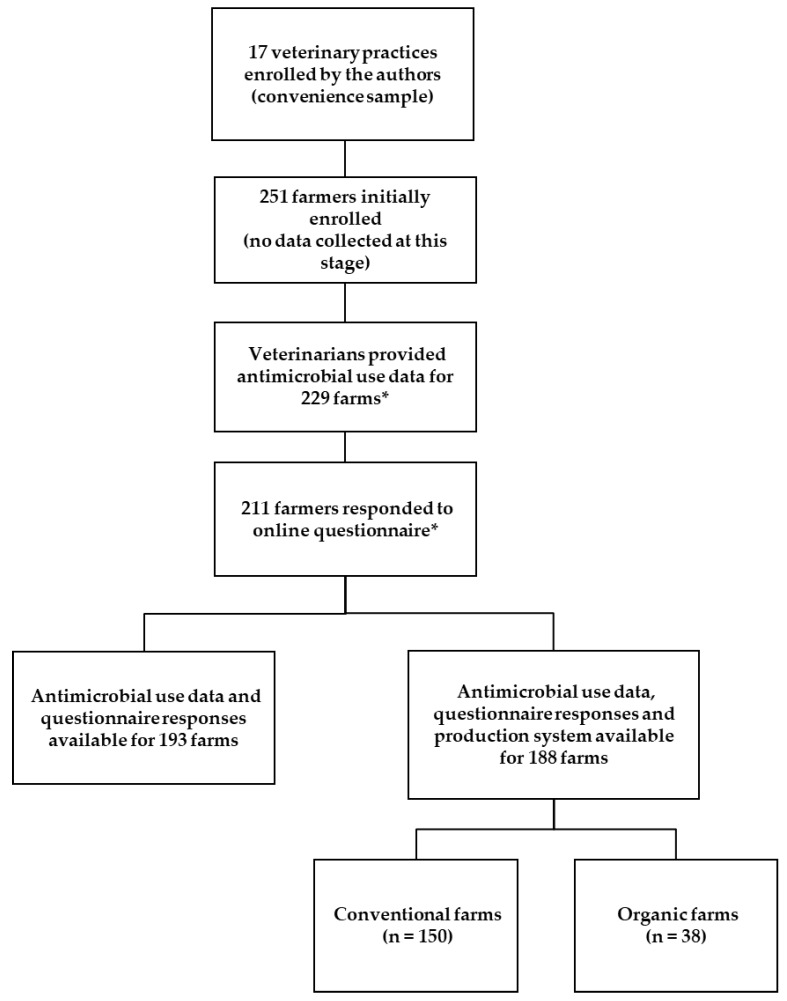
Flow chart of study participants. *These farms did not necessarily overlap; some farms provided questionnaire responses but no treatment data was available, and vice versa.

A total of 211 farmers responded to the farm management questionnaire (response rate 84.1%). The antimicrobial treatment data included here cover 2500 dairy cows and the pharmaceutical formulation coded as “dry cow tubes” only. Veterinarians provided herd treatment data for 229 farms with respect to dry cow therapy (Figure 1).

Around three-quarters (73.9%) of the 6620 dairy cows included in this study were of the Austrian Fleckvieh (dual-purpose Simmental) breed, which is the most common dairy cow breed in this Central European country [28]. Holstein-Friesians made up only 11.8% of the dairy herds included here.

Antimicrobial treatment data and questionnaire responses were available for a total of 193 farms in this study population and these farms made up the basis of the analysis of dry cow therapy presented here. The population for the comparison of production system was made up of 188 farms, of which 150 (79.8%) were managed conventionally and 38 (20.2%) organically (Figure 1).

**Figure 2:**
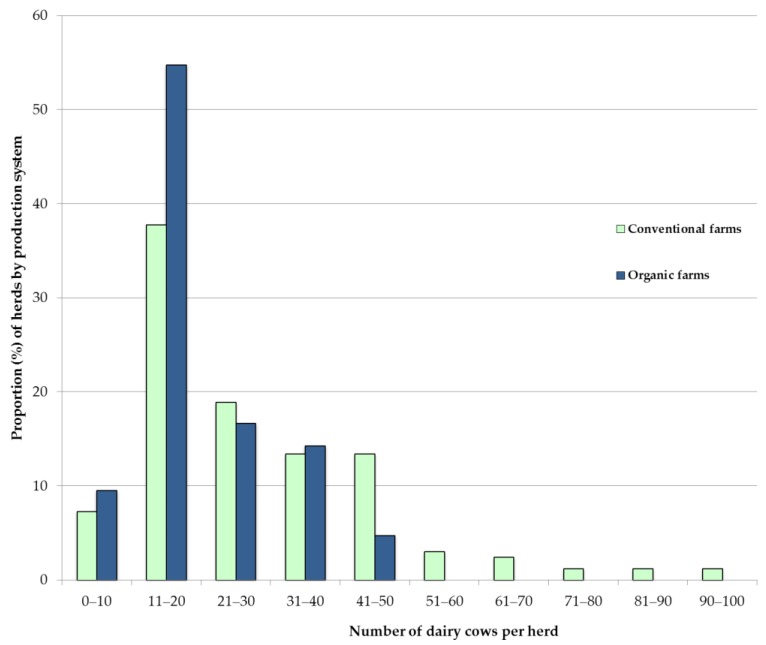
Herd size distribution (%) of conventional (n = 150) and organic (n = 38) farms.

As is common in Austria, dairy herds were predominantly small in both production systems as shown in Figure 2. The mean number of dairy cows per herd was 28.9 (median 23.0) on conventional farms and 21.6 (median 19.5) on organic farms. Conventional herds ranged from 9 to 94 dairy cows, whereas organic farms ranged from 9 to 45 dairy cows.

### 3.2. Antimicrobial Dry Cow Therapy—Questionnaire Responses

**Figure 3:**
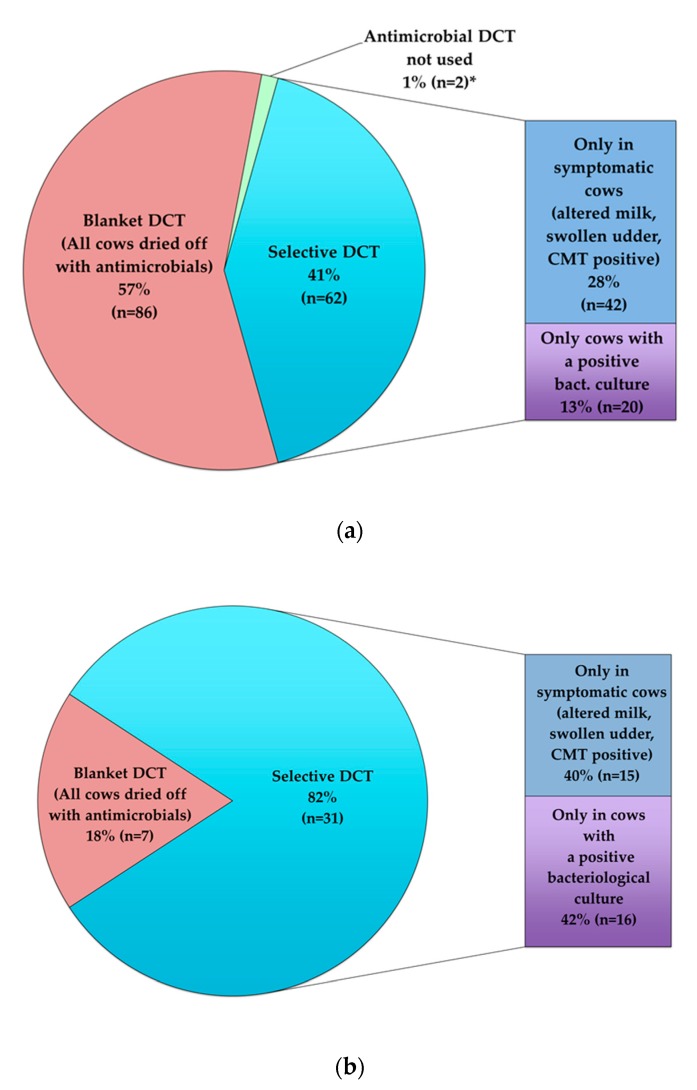
(**a**) Responses to questionnaire—conventional farmers (n = 150). (**b**). Responses to questionnaire—organic farmers (n = 38). *NB. Two farmers stated in their response to the questionnaire that they did not use aDCT at all, however, the veterinary antimicrobial treatment data showed that aDCT had been dispensed to these farmers during the study period and these farms were subsequently included in the “selective DCT” group for the statistical analysis. (aDCT = antimicrobial dry cow therapy).

As would be expected, due to the restrictions placed on organic farms, only 18.4% (7/38) of these questionnaire respondents stated that they regularly used antimicrobial dry cow therapy on all of their cows (blanket DCT). More than half of the conventional farmers, on the other hand, stated that they used blanket dry cow therapy (57.3%, 86/150). Nevertheless, conventional farmers were selective in their use of antimicrobial dry therapy on 41.3% (62/150) of farms, with decisions made according to the results of bacteriological culture or whether animals showed any signs of udder disease. Details are shown in Figure 3a for conventional farms and Figure 3b for organic farms.

### 3.3. Statistical Analysis of Defined Course Doses

A total of 193 farms were included in the analysis of antimicrobial use for blanket vs selective dry cow therapy, regardless of production system. Five farms were in the process of converting from conventional to organic production and were excluded from the comparison of these production systems, leaving a total of 188 farms in this population. Descriptive statistics by treatment type (blanket or selective aDCT) are provided in Table 1, and by production system in Table 2.

**Figure 4:**
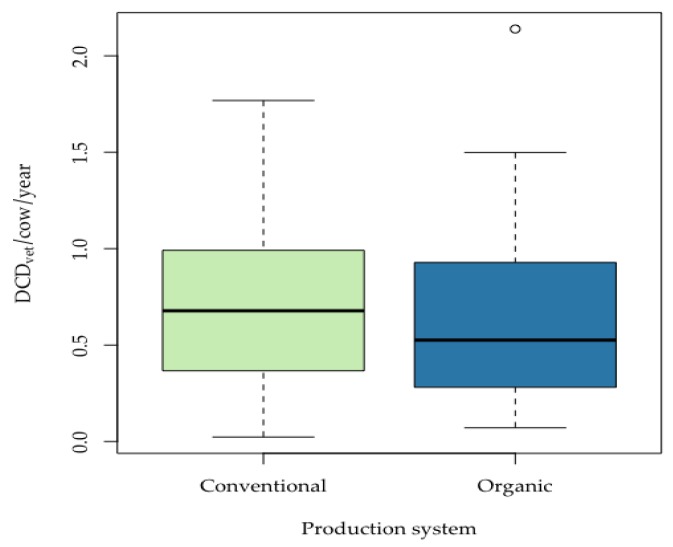
Defined Course Doses for aDCT on conventional and organic dairy farms (aDCT = antimicrobial dry cow therapy, DCD_vet_ = Defined Course Dose) Box = range between Q1 and Q3; horizontal line = median; lower whisker = Q1−1.5 (IQR) (interquartile range); upper whisker = Q3 + 1.5(IQR); circles = outliers.

There was no significant difference determined between overall antimicrobial use for DCT on conventional and organic farms with respect to the median DCD_vet_/cow/year (conventional farms: median 0.68, 95% CI (0.57; 0.80) and organic farms: median 0.53, 95% CI (0.30; 0.79); test statistic W = 3219, *p* = 0.22) (Figure 4).

**Figure 5:**
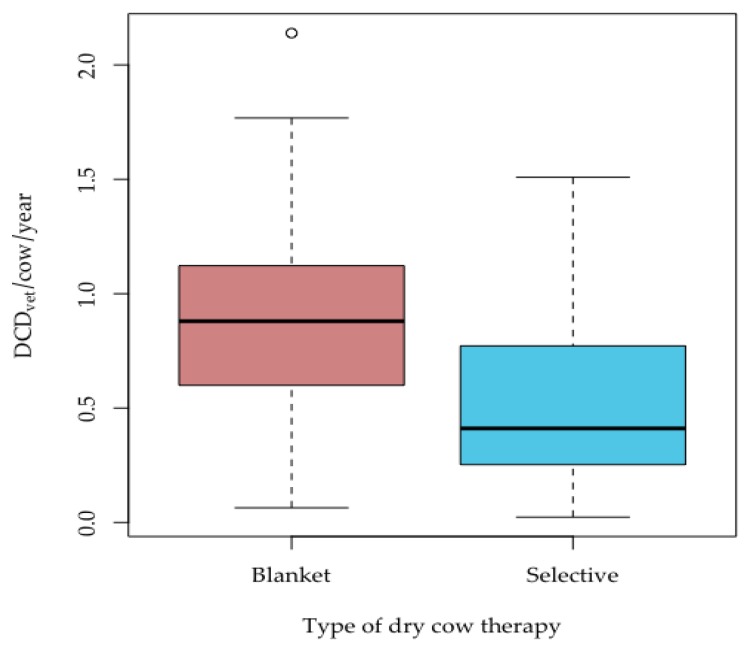
Defined Course Doses for individual herds for blanket and selective aDCT. Box = range between Q1 and Q3; horizontal line = median; lower whisker = Q1−1.5 (IQR) (interquartile range); upper whisker = Q3 + 1.5(IQR); circles = outliers.

When production system was not included in the analysis (n = 193), a statistically significant difference was, however, observed between antimicrobials used for blanket (n = 95) and selective (n = 98) DCT with respect to the median DCD_vet_/cow/year (blanket treatment: median 0.88, 95% CI (0.80; 0.97) and selective treatment: median 0.41, 95% CI (0.33; 0.54); test statistic W = 6823, *p* ≤ 0.001) (Figure 5).

Comparing all four possible treatment groups with each other, the Kruskal–Wallis test determined a significant difference between conventional or organic farms with respect to blanket or selective DCT (X^2^ = 31.09, df = 3, *p* < 0.001). The post hoc Dunn test showed a significant difference in the median DCD_vet_/cow/year between conventional–blanket DCT and conventional–selective DCT (conventional–blanket DCT n = 86, median 0.88, 95% CI (0.77; 0.95) and conventional–selective DCT n = 64, median 0.40, 95% CI (0.33; 0.52); *p* < 0.001 for both Bonferroni (B) and Benjamini–Hochberg (BH) adjustments), but no statistically significant difference could be confirmed between conventional–blanket DCT and organic–blanket DCT (conventional–blanket DCT n = 86, median 0.88, 95% CI (0.77; 0.95) and organic–blanket DCT n = 7, median 1.11, 95% CI (0.52; 1.330); *p* = 1.0 (B) and *p* = 0.33 (BH)), and conventional–selective DCT and organic–selective DCT (conventional–selective DCT n = 64, median 0.40, 95% CI (0.33; 0.52) and organic–selective DCT n = 31, median 0.36, 95% CI (0.30; 0.68), *p* = 1.0 (B) and *p* = 0.39 (BH)).

The significance of the difference between organic–blanket DCT and organic–selective DCT with respect to the median DCD_vet_/cow/year was dependent on the chosen alpha-adjustment method (organic–blanket DCT n = 7, median 1.11, 95% CI (0.52; 1.33) and organic–selective DCT n = 31, median 0.36, 95% CI (0.30; 0.680); *p* = 0.11 (B) and *p* = 0.03 (BH)). It is important to note that the organic–blanket DCT group was very small (n = 7), and the subsequent low statistical power reduces the reliability of this part of the analysis.

## 4. Discussion

The analysis presented here compared the magnitude of antimicrobial treatments under two different dry cow therapy strategies (blanket or selective DCT) on conventional and organic dairy farms in Austria. There are many different methods of quantifying antimicrobial use and no one metric is ideal for all situations [29,30]. As dry cow therapy involves the use of one application of long-acting antimicrobials, the European Medicine Agency suggests the use of Defined Course Doses (DCD_vet_) rather than Defined Daily Doses (DDD_vet_) [31]. The EMA definition of the DCD_vet_ for dry cow therapy is as follows: “the Defined Course Dose is the assumed average dose per animal per treatment course” and for dry cow products 1 DCD_vet_ = 4 intramammary injectors [31].

The prophylactic use of antimicrobials for dry cow therapy in all cows of the herd is not recommended in Austria but is not legally prohibited [32]. Treatment decisions and the control of antimicrobial substances remain in the hands of the treating veterinarians. Antimicrobial substances can only be dispensed to farmers who are members of the Animal Health Service (AHS) and who have completed the relevant medication courses. If farmers are not members of the AHS, then veterinarians have to administer each antimicrobial treatment themselves, even intramammary injectors. While controls are relatively strict in Austria compared to many other countries, Scandinavian countries are even more restrictive in their antimicrobial use [33]. In Denmark, for example, aDCT is only permitted if clinical mastitis is diagnosed within 30 days prior to drying off or if a mastitis pathogen is cultured within this period [16]. Furthermore, on Danish organic farms, only veterinarians are allowed to treat cows with antimicrobials and the withdrawal period after treatment is required to be tripled by local organic milk processors compared to the standard withdrawal period for conventional herds [16].

A study of 152 organic farms in Switzerland reported that aDCT was regularly used on 65.3% of these farms, however, it was not stated whether this was blanket DCT of all cows regardless of udder health status or whether the farmers questioned were only treating cows with previous udder disease (selective DCT) [21]. This Swiss study also stated that levels of antimicrobial-resistant bacteria (particularly *Staphylococcus aureus* and coagulase negative staphylococci, CNS) in mastitis milk samples on these organic farms were detected at a similar level as antimicrobial resistance rates in these bacteria on conventional Swiss farms [21].

A survey completed by 98 farmers in Germany reported that blanket DCT was still used on 79.6% of dairy farms in 2014, with bacteriological culture of milk prior to drying off done in at least some cows on 31.0% of farms, and in all cows on only 6.6% of farms [34]. Similarly, a French survey of 24 farmers reported that the majority (58.3%) of those questioned used blanket DCT, with only organic farmers (5/24) consistently stating that they were more selective in using antimicrobial DCT [35]. By comparison, a recent study of 715 dairy farmers in Finland reported that 78% used antimicrobials selectively at drying off, with only 13.3% of these farmers using blanket DCT [17]. While the Finnish results were primarily for conventional farms (only 2.2% of the surveyed farmers were organic producers), the low number of farmers stating that they used blanket DCT is expected in Scandinavian countries, regardless of production system, due to their strict antimicrobial use restrictions [17]. A recent Austrian study reported that of 88,534 dairy cows from 1657 farms, only 31.3% (27,723 cows) received aDCT [36]; it is important to note, however, that this study reported antimicrobial use at drying off by cow, rather than farm, and did not analyse production type. Bacteriological culture is frequently carried out in Austria, as these diagnostic tests are often provided without charge or heavily subsidised for members of the Animal Health Service. As a high proportion (80.5% in 2017) of Austrian dairy farmers are also enrolled in the national milk recording scheme [28], a large amount of data is available to the individual farmer with which to make dry cow therapy decisions.

To the authors’ knowledge, the only data currently published on antimicrobial dry cow therapy using the Defined Course Dose units, as defined by the European Medicines Agency, are in the form of epidemiological data for the national dairy herd of Ireland [26]. More and colleagues reported intramammary antimicrobial use based on national sales data rather than individual farm data. They also corrected their data by the national mean calving interval of 398 days and mean annual replacement rate of 20.4% [26]. The data presented here, therefore, differ somewhat in that we had access to individual mean calving interval and replacement rate data for each farm from the Federation of Austrian Cattle Breeders (ZAR) and each DCD_vet_/cow/year was corrected according to these factors for each individual farm. Nevertheless, the Irish authors reported an estimated on-farm DCD_vet_ per 1000 animals per year of 1022 (i.e., 1.022 DCD_vet_/cow/year) in 2015 [26], which is slightly higher than the median DCD_vet_/cow/year of 0.88 (mean 0.86) based on the treatment data from the Austrian farms included here. The Irish study did not state whether sales were for the administration of antimicrobials to cows on organic or conventional farms. A Dutch study also reported antimicrobial use for dry cow therapy, however, these data are not provided in DCD_vet_ units, but in the Dutch national unit of ADDD (daily doses per cow and year) [6]. The Dutch data are not, therefore, directly comparable with either our Austrian data or the published Irish data, although they are adjusted for calving interval and replacement rates. The mean number of daily dosages per cow per year for dry cow therapy on 94 Dutch farms from 2005 to 2012 was 2.57 ADDD; according to the authors, this means that on average 2.6 udder tubes per cow were used for blanket dry cow therapy [6]. The European Medicines Agency states that four intramammary injectors (udder tubes) are equivalent to one DCD_vet_ for dry cow therapy [25]. As such, the mean DCD_vet_/cow/year reported here in Austria, namely 0.86 for blanket and 0.54 for selective DCT, regardless of production system, is approximately equal to the use of 3.4 intramammary injectors per cow and year for blanket DCT and 2.2 intramammary injectors per cow and year for selective DCT.

It is important to note that this study was based on a convenience sample of dairy farmers and of veterinarians interested in the use of veterinary antimicrobials. The results of this study cannot, therefore, be extrapolated to the remainder of the country. However, the herd structure, proportion of cattle breeds and production systems were comparable with those of the national average, as reported previously [22,23]. In the dairy cow population studied here, a separate analysis of milk samples sent to farmers’ local laboratories, as well as culture results from our university laboratory, determined that the most common causes of mastitis over this observational period (3020 quarter milk samples from 647 dairy cows, 85.5% of which were taken only once from one cow) were staphylococci (49.7% of all samples with bacterial growth, primarily *Staphylococcus aureus* and CNS), followed by streptococci (27.8% of all samples with bacterial growth, primarily *Streptococcus uberis* and *Streptococcus dysgalactiae*) [37].

The study was not statistically powered to determine a difference between conventional and organic farms as this information was not available at enrolment but was provided by the farmers themselves as part of the online questionnaire. The organic group was relatively small and no clear conclusions can be drawn on the apparent differences in antimicrobial use for dry cow therapy determined in this study population between conventional and organic farms. However, it is surprising that organic farms in this study appear to have used antimicrobials for DCT at a higher level (expressed in DCD_vet_/cow/year) than conventional farms, despite being subject to restrictions on medicine use under both Austrian and EU organic regulations, which prohibit prophylactic use and the use of more than three medicinal treatments per dairy cow per year.

These antimicrobial use data collected over a calendar year and combined with herd data such as calving interval, replacement rate and diagnoses, as well as farmers’ management decisions, provide a useful insight into the use of antimicrobial substances for dry cow therapy in Austria. Of particular interest is the fact that there was a statistically significant difference in antimicrobial use expressed in nDCDvet/cow/year by farmers stating that they carried out blanket DCT of all cows in their herd compared to those who were more selective in their use of antimicrobials. As such, this analysis of treatment data confirmed the questionnaire responses given by the farmers regarding their antimicrobial use for dry cow therapy. This demonstrates that selective DCT is beneficial to prudent use of antimicrobials programmes and should be encouraged and supported to allow both farmers and veterinarians to make informed decisions about their antimicrobial use. Nevertheless, to ensure optimal animal health and welfare, it is essential to consider the whole farm management situation, the bacterial population present and overall milking and farm hygiene, as well as the immune system of the individual cow, when making decisions about whether to be more selective in the use of antimicrobial dry cow therapy.

## Figures and Tables

**Table 1:** Antimicrobial dry cow therapy by treatment type, based on antimicrobial data from veterinary practice software (n = 193). DCT = dry cow therapy, DCD_vet_ = Defined Course Dose

Treatment Type	No. of Farms	Corrected DCD_vet_/Cow/Year					
		Mean	Minimum	25th Percentile	Median	75th Percentile	Maximum
Blanket DCT	95	0.86	0.06	0.60	0.88	1.12	2.14
Selective DCT	98	0.54	0.02	0.25	0.41	0.77	1.51

**Table 2:** Antimicrobial dry cow therapy by production system, based on antimicrobial data from veterinary practice software (n = 188).

Treatment type	No. of Farms	Corrected DCD_vet_/Cow/Year					
		Mean	Minimum	25th Percentile	Median	75th Percentile	Maximum
Conventional farms (all DCT)	150	0.70	0.02	0.37	0.68	0.99	1.77
Blanket DCT	86	0.84	0.06	0.59	0.88	1.08	1.77
Selective DCT	64	0.52	0.02	0.25	0.40	0.77	1.51
Organic farms (all DCT)	38	0.64	0.07	0.28	0.53	0.93	2.14
Blanket DCT	7	1.04	0.20	0.69	1.11	1.23	2.14
Selective DCT	31	0.55	0.07	0.28	0.36	0.78	1.50
